# Successful treatment of relapsed Ménière’s disease using selective serotonin reuptake inhibitors: A report of three cases

**DOI:** 10.3892/etm.2013.1412

**Published:** 2013-11-15

**Authors:** FUMIYUKI GOTO, TOMOKO TSUTSUMI, KAORU OGAWA

**Affiliations:** 1 Department of Otorhinolaryngology, National Hospital Organization, Tokyo Medical Center, Tokyo 152-0021, Japan; 2 Department of Otorhinolaryngology, Keio University, Tokyo 160-8520, Japan; 3 Department of Otorhinolaryngology, Hino Municipal Hospital, Tokyo 191-0062, Japan

**Keywords:** Ménière’s disease, serotonin selective reuptake inhibitors

## Abstract

Patients with Ménière’s disease who have relapsed following endolymphatic sac surgery (EDS) or intratympanic gentamicin injection are occasionally treated with intratympanic gentamicin injections or revision surgery. However, there is a potential link between Ménière’s disease and anxiety or depression. The use of serotonin selective reuptake inhibitors (SSRIs) is likely be beneficial in the treatment of patients with Ménière’s disease. The aim of this report is to describe the benefits of SSRIs in patients with relapsed Meniere’s disease. Over the course of two years, three patients were treated for symptoms associated with Ménière’s disease with an SSRI (sertraline), with the complete resolution, or significant improvement, of symptoms. In these cases, the SSRI may have treated the associated morbidity and not Ménière’s disease itself. Ménière’s disease that appears to be resistant to typical otological treatment may not be just Ménière’s disease. Ménière’s disease may co-exist with three other conditions that are able to cause vestibular symptoms and respond to SSRIs: migraine-associated vertigo (MAV), panic disorders and chronic subjective dizziness (CSD).

## Introduction

Intratympanic gentamicin injections (1) or revision surgery (2) have been used for the treatment of patients with Ménière’s disease who have relapsed following endolymphatic sac surgery (EDS) or intratympanic gentamicin injection. Ménière’s disease is a condition with vertigo, tinnitus (ringing or buzzing noises in the ears) and progressive deafness. Ménière’s disease is caused by a dysfunction of the endolymphatic sac in the inner ear. It is an unpredictable disease that requires various types of treatment. It is estimated at approximately 1 in every 1,000 individuals suffers from Ménière’s disease. The disease may develop at any age, but more commonly does so when the patient is aged between 40 and 60 years old. With respect to the treatment, the conservative therapy is the first choice. Gentamicin injection and surgery are only performed to the patients with intractable cases. The success rate of either treatment is relatively high and up to 90% of the patients may control their symptoms. The gentamicin injection may be performed without hospital admission, but the surgery requires hospitalization. There is a risk of hearing impairment following gentamicin injection and success of either treatment have been previously reported (2,3). Thus far there has only been one study concerning the treatment of Ménière’s disease by SSRIs (3). A potential link between Ménière’s disease and anxiety or depression, and the use of serotonin selective reuptake inhibitors (SSRIs) in patients with Ménière’s disease was first reported by Overton (3). Over two years, we treated three patients for symptoms associated with Ménière’s disease with an SSRI (sertraline), with either the complete resolution of the disease or a significant improvement in the symptoms. Ménière’s disease, which has uncertain etiology and multiple ineffective treatments, warrants a clinical trial assessing the efficacy of SSRIs, as evidence from 1861 to the present day links Ménière’s disease with stress, sympathetic stimuli and labyrinthine microcirculation (3–5). The objective of this report is to describe the benefits of administering SSRIs to patients with relapsed Ménière’s disease. The written informed consent was obtained from the patients.

## Case reports

### Case 1

A 59-year-old female first presented at the Hino Municipal Hospital (Tokyo, Japan) following a relapse in spells of vertigo 15 years following EDS (Table I). The patient’s vertigo attacks usually lasted several hours, with nausea and a worsening of tinnitus, and occurred a few times a week. Careful analysis of the patient’s history revealed marked anxiety regarding the vertigo attacks, although the patient’s Hospital Anxiety and Depression Scale (HADS) score revealed no significant anxiety. The patient had no history of migraine or a present migraine. Sertraline (50 mg/day) therapy was commenced. The patient’s vertigo spells as well as anxiety improved for two weeks and stabilized. The symptoms of Ménière’s disease did not recur. Sertraline treatment ceased after six months. The patient’s functional level scale (FL) score (6) improved from 4 to 1, and the patient remained symptom-free a year later.

### Case 2

A 45-year-old male experienced a relapse in vertigo spells seven years after intratympanic gentamicin treatment. The patient had no history of anxiety disorders or migraines, past or present. The patient feared a relapse of Ménière’s disease and wanted to be a candidate for EDS. Although the patient’s HADS score was within the normal range, the patient’s anxiety regarding the attacks was marked. The vertigo attacks occurred several times a week for ~1 h. The patient was persuaded to undergo treatment with sertraline (50 mg/day) for two weeks prior to surgery. The vertigo spells were completely controlled. Sertraline treatment was continued for six months and, a year later, the symptoms remained well controlled.

### Case 3

A 72-year-old female first presented at the Hino Municipal Hospital following a relapse in vertigo spells 18 years after EDS (Table I). The patient had no history of anxiety disorders but did have a migraine headache. The vertigo attacks had occurred three times a week for a month and were usually accompanied by headache. The patient was afraid of a relapse of Ménière’s disease. However, from the patient’s clinical history, migraine-related vertigo was suspected and sertraline (50 mg/day) therapy was initiated. Following two weeks of treatment, the vertigo and headaches were controlled. Sertraline treatment was continued for six months, and the patient remained vertigo-free a year later.

In all three patients, vertigo attacks were completely controlled by the administration of sertraline; however, the hearing thresholds of the patients did not alter.

## Discussion

It is difficult to determine whether the patients in the present study were exhibiting Ménière’s disease itself; the etiology was similar to that of Ménière’s disease, but was well treated by SSRIs. The key for differential diagnosis is to consider the clinical history of the patient very carefully. The characteristic symptom of Ménière’s disease is rotating vertigo attacks usually lasting up to several hours (3). Ménière’s disease may co-exist with other conditions that cause vestibular symptoms and are responsive to SSRIs. These include migraine-associated vertigo (MAV), panic disorders and chronic subjective dizziness (CSD) (7). The diagnosis of MAV (8) is likely in Case 3, but the other two patients had no history of migraines. However, MAV may cause episodes of rotary vertigo, indistinguishable from the vertiginous symptoms of Ménière’s attacks. MAV may also cause unilateral aural symptoms (fullness and tinnitus) during attacks (8).

Panic disorders (9) are unlikely in the patients in the present study, given that the diagnostic criteria of a panic disorder include a discrete period of intense fear or discomfort developing abruptly and reaching a peak within 10 min. However, in Cases 1 and 2, a situation similar to a panic disorder is suggested by the response to the SSRI. CSD is unlikely, as CSD is defined not as vertigo attacks but as ‘persistent (≥3 months) sensations of nonvertiginous dizziness, or subjective imbalance present on most days’ (7). However, it is difficult to determine whether the information derived from the subjective complaints of the patients is accurate. A study by Boleas-Aguirre *et al*(10) revealed that anxious patients with Ménière’s disease had a high likelihood of developing CSD-like symptoms following gentamicin treatment even if they had excellent vertigo control. The benefit of SSRIs in patients with CSD has been reported (11), but there are few reports concerning the use of antidepressants in patients with Ménière’s disease. A previous study reported that antidepressants are useful in the treatment of headaches in patients with Ménière’s disease (12).

Levels of depression or anxiety in patients with relapsed Ménière’s disease are not easily evaluated, and a subjective scale such as HADS may not accurately indicate patient mood changes. SSRIs may be a therapeutic option for the relapse of Ménière’s disease following intensive therapy. Further study is required to establish the efficacy of the therapy.

In conclusion, the three cases of relapsed Ménière’s disease were successfully treated with an SSRI. However the SSRI may have treated the associated morbidity and not Ménière’s disease itself. Ménière’s disease that appears to be resistant to typical otological treatment may not be Ménière’s disease alone.

## Figures and Tables

**Table I:** Summary of cases.

Case	Side	Gender	Age (years)	PTA (dB)		Migraine	Prior to SSRI treatment			Following SSRI treatment		
		
				Right	Left		FL	DHI (P,E,F)	HADS (A,D)	FL	DHI (P,E,F)	HADS (A,D)
1	Left	Female	59	18.8	50.0	No	4	60 (12,26,22)	9 (6,3)	1	0 (0,0,0)	8 (5,3)
2	Right	Male	45	41.3	12.5	No	4	60 (10,28,22)	8 (5,3)	1	0 (0,0,0)	7 (4,3)
3	Right	Female	72	41.3	18.8	Yes	4	54 (12,22,20)	8 (5,3)	1	22 (4,8,10)	7 (4,3)

Hearing threshold was calculated as an average of four consecutive frequencies of 0.5, 1, 2, and 4 kHz. SSRI, selective serotonin reuptake inhibitor, PTA, pure tone audiometry; FL, functional level scale score; DHI, dizziness handicap inventory; P, physical; E, emotional; F, functional; HADS, Hospital Anxiety and Depression Scale; A, anxiety; D, depression.
